# Knowledge, Attitude, and Practice of Nurses Working in the Adult Intensive-Care Unit and Associated Factors towards the Use of Physical Restraint in Federally Administered Hospitals in Addis Ababa, Ethiopia: A Multicenter Cross-Sectional Study

**DOI:** 10.1155/2021/5585140

**Published:** 2021-05-24

**Authors:** Lielt Mersha Woldekirkos, Tilahun Jiru, Heyria Hussien, Belayneh Shetie

**Affiliations:** ^1^School of Nursing, College of Medicine and Health Science, Departments of Emergency and Critical Care Nursing, University of Gondar, Gondar, Ethiopia; ^2^Department of Emergency Medicine, School of Medicine, College of Health Sciences, Addis Ababa University, Addis Ababa, Ethiopia

## Abstract

**Background:**

Physical restraint is any chemical or physical involuntary method restricting an individual's movement, physical activity, or normal access to the body. Physical restraints are prescribed by the physician, but the ICU nurse remains the decision maker responsible in assessing the need, application, and removal of PR on patients in the ICU setting.

**Objectives:**

This cross-sectional descriptive study was carried out to determine the knowledge, attitudes, and practices of nurses working in adult ICU and associated factors towards the use of physical restraints in federally administered hospitals in Addis Ababa, Ethiopia, 2019.

**Methods:**

The study was conducted in ICUs of Federal Hospitals in Addis Ababa, Ethiopia, 2019. A hospital-based descriptive cross-sectional study design was carried out. By census, a total of 126 nurses were included. The data were checked for their completeness and were entered to EpiData version 4.2 and analyzed using SPSS version 25 software with 95% CI. Also, the Pearson correlation coefficient and binary logistic regression analysis were used to find an association.

**Result:**

Majority of nurses was found to be aged between 21 and 30 years, (62.5%) have worked 2–5 years, and (83%) were degree graduates. The nurses' knowledge score was 6.1 ± 2.6 (50.8%) with possible range 0–11, the attitude score was 14.1 ± 3.1 (64%) with possible range 0–22, and the practice score was 13.9 ± 3.8 (63.18%) with possible range 0–22. Their demographical characteristics such as gender, working year, and education levels were not significantly associated with knowledge, attitudes, and practices (*P* > 0.05). Only age significantly associated with practice. Lack of a written policy or guideline and not being trained on application of physical restraint were significantly associated with knowledge. Also, practice was associated with knowledge and attitude.

**Conclusion:**

According to the study, there was a poor level of nurses' knowledge, proper attitude, and satisfactory practice toward the use of physical restraints.

## 1. Introduction

Critically ill patients are characterized by having life-threatening illnesses or injuries which need continuous monitoring and intensive care. As a result, they are attached to life support and monitoring equipment. Since they may harm themselves unintentionally by removing end tracheal tubes, taking out vascular access, arterial lines, or monitoring equipment, they need protection to ensure their safety [1]. One of the most common practices in the ICU to insure the patient's safety is physical restraint. Physical restraint is any chemical or physical involuntary method restricting an individual's movement, physical activity, or normal access to the body [2]. According to Professional Development Committee of the Nursing Council of Hong Kong (PDCNC) definition, physical restraint (PR) is any device, material, or equipment attached or adjacent to an individual's body that he/she cannot easily remove, thus immobilizing or reducing the ability of the individual to move his/her body parts freely and/or to have normal access to his/her own body [3].

Physical restraint is a heavily debated procedure because of the questionable ethical and legal issues affecting autonomy and dignity of patients [4]. However, health professionals use physical restraint (PR) as a last resort when alternative measures failed and a patient's safety is at risk, for instance, a patient at risk of falling; hurting themselves or others; pulling out tubes; or acting in an aggressive or violent way [5]. Except in emergencies, individual decisions regarding restraint should be discussed within the multidisciplinary teams, with the involvement of the individual and their family as far as possible [6].

According to Joint Commission Standards on Restraint and Seclusion/Nonviolent Crisis Intervention Training Program, restraint or seclusion should be used only when it can be clinically justified or when warranted by patient behavior that threatens the physical safety of the patient, staff, or others. Also, the hospital uses the least restrictive form of restraint that protects the physical safety of the patient, staff, or others [7]. Written policies and procedures are needed that guide the use of restraint, evaluate and reevaluate the patient who is restrained or secluded, document the use of restraint or seclusion, and train staff to safely implement the use of restraint or seclusion [7, 8].

Physical restraint use in ICUs has a long history throughout the world. While European countries such as England and France reacted to physical restraint in the nineteenth century, it was widely used as an ethical and appropriate therapeutic measure in the United States. Moreover, it was used in 1980 in ICUs and medical-surgical wards [9], while other countries, such as the UK and Norway, consider restraints to be unacceptable [2].

There are many forms of physical restraints, including wrist, ankle, chest, and waist [1]. According to a study in Portugal conducted on physical restraint application, the most commonly used materials were the bands, linen, and cotton, and most participants highlight the wrists and chest as parts of the body to immobilize [10]. In a similar study in Egypt, the most commonly used type of physical restraint involved restraining the upper and the lower limbs followed by bilateral wrist restraints and then bedside restraints. Gauze and dressings were the types of restraint materials commonly used in both shifts [11]. In South Africa, the commonest types of restraints used were bed rails 93% and wrist belts 12%. Restraints were used largely to protect medical devices and as protection from harm [12].

**Figure 1:**
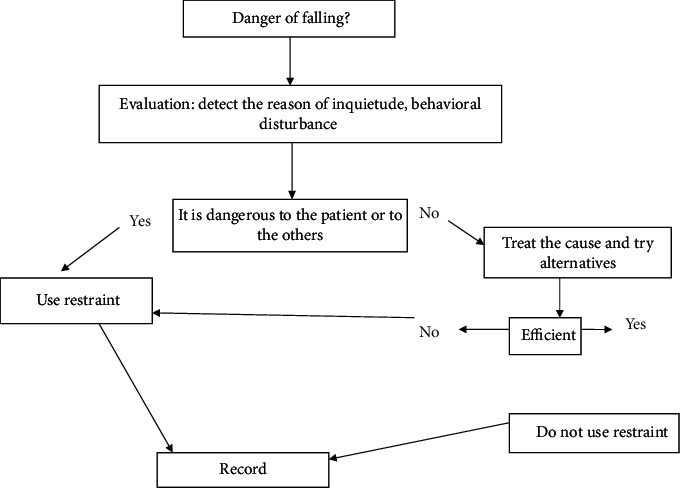
Decision-making algorithm for physical restraint [13].

Also, according to the 2015 Liverpool Hospital ICU Guideline, nursing staff may need to restrain a patient in order to protect the patient from injury, protect themselves from unnecessary risk or harm, and prevent removals of vital treatment modalities. Therapeutic restraints: if all alternative measures have been considered and implemented and are unsuccessful, then the use of physical restraints may be considered [8] (Figure 1).

In a systematic review of 52 articles, Rose et al. (2016) found that physical restraints were used to prevent removal of the endotracheal tube as well as nasogastric tubes, urinary catheters, and central lines which are needed in an ICU environment as these measures provide life-saving treatment [14, 15]. A study conducted in Malaysia in 2016 showed that the most common reported reason was “trying to pull out tubes and catheters and prevent fall” [16].

Barriers to shortening the restraint use included fear of patient injury, staff and resource restrictions, lack of education and information about alternatives to restraints, policy and management issues, beliefs and expectations of staff, family, and patient, inadequate review practices, and statement barriers [2].The availability of clear and standardized guidelines in the form of a policy document for the professionals is, therefore, crucial in supporting them in such ethical and legal dilemmas [17].

Researchers showed that the rate of physical restraint use in ICUs is 24%–40% times more than in general hospital wards [9]. According to a study conducted in Europe, the prevalence of PR was 60% [18]. According to a meta-analysis study in Iran, 2017, the prevalence of physical restrains use was estimated to be 46.7% [19]. However, in some countries, it has low prevalence. According to an observational study in Israel, the prevalence was 3.59%. This positive result may be due to different reasons. Among that, in Israel, physical restraint use is regulated by the Ministry of Health regulation [20]. In Malaysia, a study in 2016 showed 19.7% [16]. A similar finding by a descriptive study in South Africa showed 23% [17]. PR is commonly practiced in the US, Australia, and Europe. A study conducted in Portugal showed that 92.3% professionals consider the physical restraint is a way of ensuring the patient's safety [13].

Despite great tendency toward its use for ensuring patient safety, physical restraint has been reported to be associated with negative and harmful effects such as pressure ulcer, depression, severe life-threatening injuries, and finally, death [9]. One hundred deaths in the USA occur annually due to injuries by improper physical restraint [2]. According to a study conducted in Europe, restrained patients were hospitalized twice as long as those who were not restrained, and the mortality increased in those patients who were restrained [18]. Complications reported by researchers include edema and cyanosis by wrist and arm restraints, pressure ulcers, and aspiration and breathing problems caused by sheet and belt pressure on the chest, head hits by angry patients on bed sides, contractures of joints, and rejecting meals. Asphyxiation is the most common cause of restraint-related death [2].

Physical restraint use in hospitals has been studied in detail in different countries such as the Asian [4, 21], European [13, 22], and African countries [1, 11, 23]. Even though it is a common practice in Ethiopia, to the best of our knowledge, still there is no any study conducted on it. This study is the first to examine the knowledge, attitude, practice, and associated factors of nurses working in the adult ICU on the use of restraint in Ethiopia. So, knowing KAP of health workers and factors associated is necessary before developing policies and guidelines to provide quality care improvement in hospitals. In Ethiopia, according to our observation, physical restraint is a more conventional and common practice in ICUs, ED, psychiatric department, and at home traditionally without any guidelines or policies. For this practice, still there is no enough research conducted on it.

## 2. Materials and Methods

### 2.1. Study Area and Population

A multicentre institution-based cross-sectional study was conducted among nurses working in the adult ICU in Addis Ababa federally administered hospitals from March 11 to April 25, 2019 in Addis Ababa, Ethiopia. Addis Ababa is a capital city of Ethiopia with an attitude of 2300 m above sea level. It is commonly known as the capital city of Africa as a result of many head quarters of different international and regional organizations such as the Africa Union and UN Economic Commission for Africa found in the city. In Addis Ababa, there are 37 hospitals, two NGOs, twelve governmental organizations, and twenty-three private hospitals. Out of twelve governmental hospitals, ten of them have an ICU. There are four federal hospitals; they are, Black Lion Hospital, St.Paulos Hospital, St Petros Hospital, and Alert Hospital. The study population was all nurses who working in adult ICUs in the four federal hospitals in Addis Ababa, Ethiopia. Nurses who were on vacation, sick leave, and annual leave during data collection and with work experience less than six months were excluded from the study. For sampling, by census, all nurses were included due to their small number. There was no need of sample size determination because the total population was taken. All nurses (126) in federally administered public hospitals of Addis Ababa (AaBET = 36, TASH = 41, ALERT = 28, and St. Petro = 21) were included in the study.

### 2.2. Data Collection Method and Instrument

The data were collected by using a self-administered semistructured questionnaire which consists of four parts ([1, 4, 21]).

  Part-1: a sociodemographic designed questionnaire was used to collect the demographic data related to nurses in the study
  Part-2: a structured designed questionnaire was used to collect the nurses' knowledge related to physical restraint, which consists of multiple-choice questions; each question has 2 choices (yes and I do not know or no), and only one is correct
  Part-3: a structured designed questionnaire was used to collect the nurses' attitude related to physical restraint, which consists of multiple-choice questions; each question has 3 choices (agree, disagree, and nondecided), and only one is correct
  Part-4: a structured designed questionnaire was used to collect the nurses' practice related to physical restraint, which consists multiple-choice questions; each question has 3 choices (always, sometimes, and never), and only one is correct

The data were collected by trained Bsc nurses under supervision from March 11 to April 25, 2019 at the selected hospital's ICU in Addis Ababa, Ethiopia. Data quality was assured before, during, and after the data collection process. Before data collection, an objective-based and standardized English-version checklist was prepared. Training was given for the supervisor and data collectors on sampling procedures and the data collection process. During data collection, there was a close day-to-day supervision in the data collection process.

#### 2.2.1. Scoring System

Part 2: nurses` knowledge regarding physical restraints among critical-care patients.

There are 12 items which include 10 correct questions and 1 false question. Different responses were scored as follows: 1 = yes and 0 = I do not know or no.

- The maximum score is 12 (1 *∗* 12) (respondents remained positive (i.e., yes) to the positive statements), and the minimum score is 0 (0 *∗* 12) (respondents in this category remained negative (i.e., no))
- Poor level of knowledge: it represents 0–6 (less than 50%)
- A fair level of knowledge: it represents 7–9 (from 50 to 75%)
- Good level of knowledge: it represents 10–12 (more than 75%)

Part 3: nurses` attitude regarding physical restraints among critical-care patients.

There are 11 items which include 9 correct questions and 2 false questions scored by the three-point Likert scale. Different responses were scored as follows: 2 = agree, 1 = nondecided, and 0 = disagree.

- The maximum score is 22 (2 *∗* 11) (respondents remained positive (i.e., agreed) to the positive statements), and the minimum score is 0 (0 *∗* 11) (respondents in this category remained negative (i.e., disagreed))
- Proper Attitude: it represents 14–22 (more than 60%)
- Improper attitude: it represents 0–13 (less than 60%)

Part 4: nurses` practice regarding physical restraints among critical-care patients. There are 11 items which include 10 correct questions and 1 false question.

- Different responses were scored as follows: 2 = always, 1 = sometimes, and 0 = never
- The maximum score is 22 (2 *∗* 11) (respondents remained positive (i.e., always) to the positive statements), and the minimum score is 0 (0 *∗* 11) (respondents in this category remained negative (i.e., never)
- Unsatisfactory practice: it represented 0–11 (less than 50%)
- Satisfactory practice: it represented 12–16 (from 50 to 75%)
- Good practice: it represented 17–22 (more than 75%)

### 2.3. Data Analysis

The collected data were checked for its completeness and entered to epidata version 4.2 and analyzed using SPSS version 25 software with 95% CI. Frequency, percentage, means, median, and standard deviation were used to describe the data using tables and figures. In addition, the Pearson correlation coefficient and binary logistic regression analysis were used to find whether knowledge, attitude, and practice might be associated with nurses' sociodemographic characteristics practice towards physical restraint use and association between dependent variables, knowledge, attitude, and practice (all variables with *P* < 0.05). A *P* value at 0.05 was used to determine significance regarding *P* value > 0.05 to be statistically insignificant. *P* value ≤ 0.05 has been statistically significant, and the *P* value ≤ 0.001 has been highly statistically significant.

### 2.4. Ethical Clearance

Ethical clearance was secured from the Research Ethics Committee (REC) of the Public Health Department as mandated by Addis Ababa University. Letter of permission was obtained from the administration officials of the hospitals. Informed consent was obtained from the selected hospital's ICU nurses prior to proceeding data collection. This was carried out after clear description of the objectives of the study and of its procedures. Then, each respondent was asked to check whether information provided on the purpose of the study was adequately understood or not. Confidentiality of the information obtained from each participant was preserved.

## 3. Result

### 3.1. Sociodemographic Characteristics

Of the total sample size which is 126, the total number of respondents who were included in the study was 112, with a response rate of 96.5%; some responses were discarded from the study because of lack of completeness, and others did not include inclusion criteria.

The age of nurses participating in the study ranged from 21 to 60 years, and the mean age rate was 27.39 (±4.23); 50.9% were male, 64.3% were unmarried, and 94(83.9%) had a bachelor's degree. Most participants, 70 (62.5%), had a work experience of 2–5 years, and their average monthly income was 5098.21 (Table 1).

### 3.2. Knowledge regarding Physical Restraint Use

A total of 112 nurses, 79 (70.5%), use physical restraint in their unit. Among them only, 34 (30.4%) have a written policy and 33 (29.5) reported having received training on application of physical restraint. More than half of the participants knew the reason of using physical restraint, 61 (54.5%) answered the question whether “physical restraint allowed protecting patients or others from injury.” They knew the complications of physical restraint; from the total, 70 (62%) answered that chocking may happen if patient restrained while lying flat in bed and 66 (58%) had information on recording physical restraint. 71 (63.4%) nurses knew that physical restraint is not the only option to calm patients. Sedation is the commonest alternative for physical restraint that was 47 (42%).On the contrary, a majority of nurses answered the following items about physical restraint as follows: of the total, 70 (62.5%) did not know about legal punishment of inappropriate use of physical restraint, more than half, 60 (53.6%), did not have time limitation, 59 (52.7%) did not examine restrained patients fluently, and 62 (55%) answered that confusion and disorientation are a good reason for physical restraint (Table 2).

There were 12 knowledge questions about PR, and the level of knowledge of nurses was calculated out of 12. The mean score and standard deviation of knowledge of the nurses working in adult ICUs on the use of physical restraints was 6.1 ± 2.61, with a possible range of 0–12 which is almost at the midpoint of the possible range (0–12 points).

**Figure 2:**
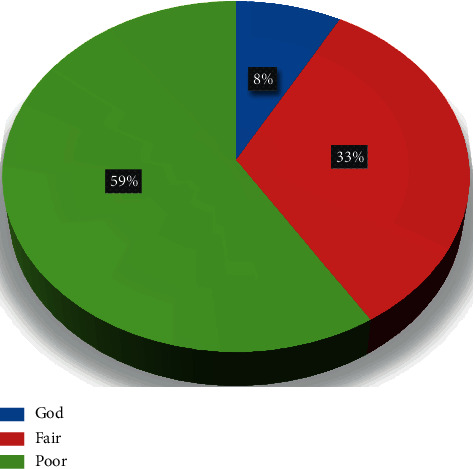
Category of nurses' knowledge regarding physical restraint (*n* = 112).

Figure 2 illustrates the level of nurses' knowledge. It was demonstrated that more than half of the nurses had a poor level of knowledge toward the application of physical restraints among critical ill patients.

### 3.3. Attitude regarding Physical Restraint Use

Regarding the attitude of nurses working in the adult ICUs towards physical restraint use, the mean score and standard deviation was 14.1 ± 3 (64%), which is slightly above the midpoint of the possible range (0–22 points). The results showed that most of the nurses answered affirmatively to the subsequent items: of the total, 67 (59%) of nurses believed that the family members have the right to refuse the use of restraints and 68 (60.7%) of nurses stated that physical restraint should be prescribed. Moreover, 60 (53.6%) participants explained that if they were patients, they felt that they have the right to refuse physical restraint, 70 (62.5%) expressed feeling discomfort when they restrained patients, 66 (58.9%) felt embarrassed when family members entered the restrained patient's room, 75 (67%) of participants believed that the hospitals are responsible to adhering to the laws of PR to ensure patients' safety, 61 (54.4%) reported that they agreed with a question “It is important to apply restraints to assure legal protection for myself and my center,” and 62 (55.4%) agreed with physical restraint may increase the risk of strangulation.” In contrast, almost half of the participants, 55 (49.1%), believe that physical restraint may decrease nursing-care time (Table 3).

**Figure 3:**
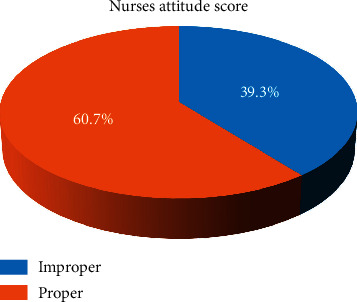
Category of nurses' attitude regarding physical restrain (*n* = 112).

Figure 3 demonstrates nurses' attitude. It represents that more than half of the nurses had proper attitude regarding to the application of physical restraints among critically ill patients.

### 3.4. Practice Regarding Physical Restraint Use

Regarding nurses' practice towards physical restraint, the mean score and standard deviation of practice was 13.9 ± 3.8, ranging from 0–22. In this section, 50 (44.6%) of participants reported that they always tried a few nursing methods before restraining the patient and 59 (52%) tried to found a reason for physical restraint. 53 (47.3%) always respond restrained patent's call for help, and 48 (42.9%) always examine the restrained patient frequently. Almost two-thirds of participants, 85 (75.9%), restrain patients when they faced staff shortage, and only 34 (30.4%) of the nurses record always the type, reason, and time of physical restraint use. In physical restraint application, the most commonly used materials were gauze, 72 (58.1%) and bed sheet, 32 (25.8%), and only 18 (14.5%) use commercially prepared belts, and most participants highlight the wrists, 83 (50.9%), ankle, 35 (21.5%), chest, 30 (18.4%), and waist, 15 (9.2%), as parts of the body to immobilize (Table 4).

**Figure 4:**
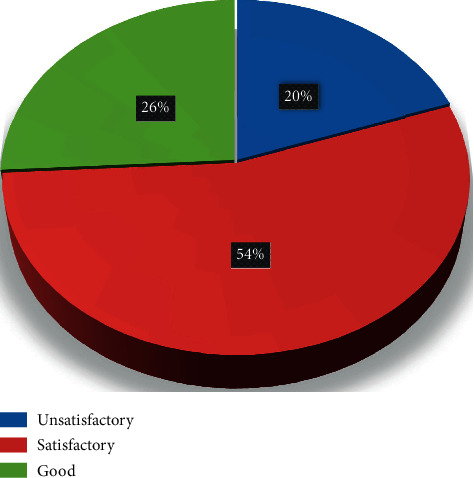
Categories of nurses' practice regarding physical restraint (*n* = 112).

Figure 4 displays categories of nurses' practice. It was found that, about 54% of nurses had a satisfactory level of practice, 20% of nurses had an unsatisfactory level of practice, and 26% of nurses had a good level of practice regarding the application of physical restraints among critical ill patients.

### 3.5. Association between Knowledge, Attitude, and Practice of Nurses towards Physical Restraint

Pearson's correlation coefficient showed there was a positive correlation between attitude (*P* ≤ 0.001), *r* = 0.34, and practice (*P* ≤ 0.001), *r* = 0.31, with knowledge of nurses towards physical restraint. Therefore, these variables were entered into the binary logistic regression model to determine which variables are associated with nurses' practice.

## 4. Discussion

### 4.1. Sociodemographic Characteristics of Participants

The current study documented that male and female were evenly distributed, that is, 50.9% and 49.1, respectively. Unlike other studies, a study conducted in Johannesburg [15] reported that a majority of the nurse respondents were females and they accounted 81.4%. Other studies in Menoufia University, Egypt, and Konya, Turkey, [1, 24] stated most of their sample were female. Regarding age, educational level, and experience years among the respondents, the study reported that the majority of participants were aged between 21 and 30 years and had a bachelor's degree in nursing and a professional experience of 2–5 years. These findings are similar with other studies conducted with nurses by M. E Maleho in Johannesburg [15], Kandeel and Attia in Egypt [11], and Kaya and Dogu in Turkey [21]. They stated that most participants had a professional experience of 1–5 years and 2–5 years. This result indicated that young and less-experienced nurses are employed in tertiary hospitals' ICUs. Regarding participants' marital status, most of them were unmarried.

### 4.2. Nurses` Knowledge about Application of Physical Restraint

In our study, in relation to physical restraint use, 70.5% of participants use physical restraint in their unit. This result is lower than that of a study in Konya, Turkey, by Balci [24]; he reported that 98% of nurses use physical restraint. By the current study, a majority of nurses did not receive any previous training about physical restraints, only 29.5% of participants had received training, and 30.4% reported that they had a guideline or a written policy for the use of physical restraint. These results are supported by the studies of El-Sol [1] and Kalula [17] in Egypt; they reported that only 13% of the nurses reported having received training as a student on the use of physical restraint, and only 39% of nurses knew of a hospital policy on the use of restraint. This implied that physical restraint is practiced without a guideline and educational background.

The present study reported that the mean of total nurses` knowledge score was (6.1 ± 2.61) 50.8% which reflected that they had a poor level of knowledge towards application of physical restraints. This finding was lower than that of other studies that revealed almost moderate level of knowledge of nurses regarding physical restraint use; the knowledge score was7.85 ± 1.86 in [1] and 7.1 ± 1.7 in [24]; in a study by Fatemeh Eskandari in Malaysia, the mean score was 40.48 (SD = 4.05) [4], and in a study by Kaya [21] in Istanbul, Turkey, the mean knowledge score of the nurses was 7.83 ± 1.59. These significant differences may be due to lack of written policy and guideline for the use of physical restraint and the nurses did not take appropriate training about application of physical restraint. Also, a majority of nurses did not understand about physical restraint in answering the following items: (53.6%) did not have a time limitation for releasing restrained patients, findings from this study were similar to those of the study by M. E. Maleho in Johannesburg, and there was no agreement about the time that an individual patient could be restrained. The period the respondents indicated ranged from “15 minutes to days.” 62.5% did not have information about legal punishment, and 55.4% answered that confusion and disorientation are good reasons to use of physical restraint (Table 5).

### 4.3. Attitude of Nurses regarding Physical Restraint Use

The current study showed that the mean score of nurses' attitude regarding use of physical restraints was 14.1 (SD = 3.1); it meant most of the nurses had a proper level of attitude related to the application of physical restraints. These findings are higher than the finding of El-Sol that was 12.23 ± 1.86; he reported that most of the nurses had an improper level of attitude. He said that high percentage of the participant nurses in his study had a negative attitude regarding the application of physical restraints among ICUs patients. It is similar with the findings of the studies of Fatemeh Eskandari (24.13 ± 3.09, 10–40) and Hatice Kaya (30.00 ± 4.82, 16–48); they reported that their result represents moderate level of nurses' attitude regarding physical restraint use, but slightly lower than that reported by Balci H. that was 31.8 (4.6).

In this study, most of the nurses agreed with the following statements evaluating their attitudes: 70 (62.5%) answered “I feel guilty placing a patient in restraints,” but in the study of Kaya [21], only 39% of participants answered this question correctly, and in the study of Eskandari [4], 25.5% answered the question. 67 (59.8%) answered “I think family members have the right to refuse physical restraint use.” In the study of El-sol [1], only 5% of nurses answered the question, and in the study of Eskandari, 22.7% answered the question. For the question “I think physical restraint should be prescribed,” 68 (60.7%) answered. 62 (55.4%) answered “I feel PR increases the risk of strangulation.”

These results indicated that the nurses had proper attitude on the use of physical restraint and they are familiar with some complications of application of physical restraint. But, in a study in Kaya, only 27.9% of nurses answered the question “I feel PR increases the risk of strangulation.”

On the contrary, a majority of nurses had improper attitude answering the following items: “I feel application of physical restraint is important to assure legal protection of self and my center”; this finding demonstrates that the participants strongly believe that physical restraint is the only and best way to protect both patients and staff from harm. Therefore, their attitude is affected by these negative thoughts and inadequate knowledge.

### 4.4. Practice of Nurses regarding Physical Restraint Use

In this, the total nurses` practice mean score regarding application of physical restraints was 13.9 (SD = 3.8, ranging from 0–22), which is 63.2% representing a satisfactory practice level; this result is in line with the studies of El-Sol [1] and Fatemeh Eskandari [4].They reported a satisfactory or moderate level of practice score of nurses, but lower than that of the study of Hatice Kaya [21], which had a good level of practice score that was 85.7%. In our study, in this section, more than half of the participants reported that they always find a reason for physical restraint before using it and only 44% of participants always tried a few nursing methods before physical restraint. Another gap identified by the current study was 33% of nurses always and 42% sometimes restrain a patient when they faced a staff shortage. A majority of the participants did not record the time and type of physical restraint on the patient card. The result showed that they practice physical restraint traditionally without considering the complications.

### 4.5. Association between Knowledge, Attitude, and Practice of Nurses towards Physical Restraint

In the use of physical restraint, the approach shown by nurses is of top priority and vital importance. So, it is considered that knowledge, attitude, and practice of nurses are interrelated and may be positively or negatively affect one another.

### 4.6. Relationship among Sociodemographic Characteristics and Nurses` Knowledge, Attitude, and Practice regarding Physical Restraints

The findings of the exciting study revealed that there was no statistical significance between nurses' educational level and knowledge, attitude, and practice; these results agreed with the study of El-sol [1]; he reported that there were nonsignificant differences in knowledge score between nurses who had a diploma and bachelor's degree in nursing (Table 5).

In our study, no significant association was found between gender, educational level and knowledge, attitudes, and practice scores of nurses. This result was similar with the study of Balci [24], but different from the findings of another study by El-sol [1]; it was found that a significant association was found between gender and knowledge and practice scores of nurses, and the knowledge level of male nurses was higher than that of female. Also, a study by Eskandari [4] showed that there was a significant association in academic qualification and knowledge of nurses towards physical restraint as degree and postbasic certified nurses showed the higher knowledge score towards physical restraint use.

By the current study, there was a statistical significance between nurses' age group with practice only. From the variables associated with practice of nurses in the bivariate logistic regression, age was statistically significant to predict practice of nurses in the multivariable logistic regression. Participants within the age group of 31–40 years are 3.6 times knowledgeable than those within the age group of 21–30, while El-sol reported that there was no statistical significant correlation between nurses' age and practice (Table 6).

The current study also found an association between nurses' knowledge and absence of a written policy and lack of training on the application of physical restraint. Participants who have not had previous training on PR had 2.6 times less knowledge than the trained ones. This result was similar with another study by M. E. Maleho in Johannesburg [15]; policy and guidelines on the use of physical restraints guide health practitioners in the management of patients (Table 7).

The current study found a statistical correlation between dependent variables. In the bivariate logistic regression analysis, the variables found to be significantly associated were practice with knowledge and attitude (Tables 8 and 9).

The study revealed that nurses with a fair level of knowledge will improve their practice level by 3 times more than those with a poor level of knowledge. Between attitude and practice, nurses with a good level of practice may have 3.9 times more proper attitude than a satisfactory and unsatisfactory level. This result is similar with the study of Fatemeh Eskandari [4]. This implied that nurses' practice level improved with proper attitude.

## 5. Conclusions

The present study concluded that there was a poor level of nurses' knowledge, proper attitude, and satisfactory practice towards physical restraints. Moreover, there was no statistical correlation between nurses' gender, educational level, year of experience and knowledge, attitude, and practice. There was a positive correlation between nurses' age and practice only; also, there was knowledge with the absence of a guideline and a written policy and lack of training. Finally, there was a statistically significant association between the dependent variables. It was between nurses' knowledge and practice score.

## Figures and Tables

**Table 1:** Personal and professional characteristics of nurses working in the adult ICU at federally administered hospitals in Addis Ababa, 2019 (*n* = 112).

Sociodemographic characteristics		Study group (*n* = 112)	
		No.	%
Age in years	20–30	97	86.6
	31–40	14	12.5
	41–50	0	0
	>50	1	0.9
Gender	Male	57	50.9
	Female	55	49.1
Qualification	Diploma	12	10.7
	Bsc degree	94	83.9
	Msc degree	6	5.4
Work experience	Less than 5 years	90	80.4
	More than 5 years	22	19.7
Type of nursing position	Duty nurse	107	95.5
	Head nurse	2	1.8
	Unit manager	3	2.7
Monthly income in ETB	1000–5000	69	61.6
	5001–10000	42	37.5
	>10000	1	0.9
Marital status	Married	38	33.9%
	Unmarried	72	64.3%
	Divorced	2	1.8%

**Table 2:** Selected items measuring participant nurses' knowledge regarding physical restraint use at federally administered hospitals in Addis Ababa, 2019 (*n* = 112).

Statements	Yes, N (%)	No, N (%)	I do not know, N (%)	Mean ± SD
Do you use physical restraints in your unit?	79 (70.5)	33 (29.5)		1.29 ± 0.458
Does your unit have a written policy on the use of physical restraint?	34 (30.4)	51 (45.5)	27 (24.1)	1.94 ± 0.74
Do you have any training on how to apply a physical restraint?	33 (29%)	73 (65.2%)	6 (5.4%)	1.76 ± 0.54
Do you know physical restraint is only allowed to protect patients or other people from injuries?	61 (54.5%)	40 (35.7%)	11 (9.8%)	1.55 ± 0.67
Do you know there may be danger of choking if a patient restrained while lying flat in bed?	70 (62.5%)	28 (25%)	14 (12.5%)	1.5 ± 0.71
Do you know restraints should be released every 2 hours, if the patient is awake**?**	53 (47.3%)	42 (37.5%)	17 (15.2%)	1.7 ± 0.73
Do you know alternatives to restraints**?**	70 (634%)	41 (36.6%)	5 (4.5%)	1.4 ± 0.58
Is there a limited time that an individual patient can be restrained in your unit?	52 (46, 4%)	45 (40.2%)	15 (13.4%0	1.67 ± 0.7
Confusions and disorientations are good reasons for the use of physical restraint	62 (55.4%)	31 (27.7%)	19 (17%)	1.62 ± 0.76
Nurses can be punished for threatening the patients if they use physical restraint when it is not required	42 (37.5%)	49 (43.8%)	21 (18, 8%)	1.8 ± 0.73
Records of usage should be kept for each patient who is restrained in every shift	66 (58.9%)	26 (23.2%)	20 (17.9%)	1.6 ± 0.78
Only in emergencies, nurses are allowed to use the physical restraint on patients without any doctor's instruction	60 (53.6%)	40 (35.7%)	12 (10.7%)	1.6 ± 0.7

**Table 3:** Selected items measuring participant nurses' attitude regarding physical restraint use at federally administered hospitals in Addis Ababa, 2019 (*n* = 112) (*n* = 112).

Statements	Agree, N (%)	Disagree, N (%)	Nondecided, N (%)	Mean ± SD
Do you think that family members have the right to refuse the use of PR?	67 (59.8%)	17 (15.2%)	28 (25%)	1.7 ± 0.85
Do you think that a PR should be prescribed by a responsible body?	68 (60.7%)	18 (16.1%)	26 (23.2)	1.63 ± 0.84
If you were a patient, do you think that you have the right to refuse being restrained?	60 (53.6%)	39 (34.8%)	13 (11.6%)	1.6 ± 0.7
Do you feel discomfort when placing a patient on restraint?	70 (62.5%)	27 (24.1%)	15 (134%)	1.51 ± 0.72
Do you feel embarrassed when family members enter the restrained patient's room when they have not been informed?	66 (58.9%)	36 (32.1%)	10 (8.9%)	1.5 ± 0.66
The hospital is responsible to adhering to the laws on the use of restraints to ensure the safety of a patient	75 (67%)	21 (18.8%)	16 (14.3%)	1.5 ± 0.74
Do you feel uncomfortable if a patient becomes more upset after being restrained?	74 (66.1%)	31 (27.7%)	7 (6.3)	1.4 ± 0.6
Do you think that placing a patient in restraints can decrease nursing-care time?	55 (49.1%)	50 (44.6%)	7 (6.3%)	1.6 ± 0.6
Patients suffer from feeling inferior when they are restrained	58 (51.8%)	39 (34.8%)	15 (13.4%)	1.6 ± 0.7
Do you think it is important to apply restraints to assure legal protection for yourself and your center?	61 (54.5%)	37 (33%)	14 (12.5%)	1.6 ± 0.7
Do you believe that restraints increase the risk of strangulation?	62 (55.4%)	39 (34.8%)	11 (9.8%)	1.5 ± 0.67

**Table 4:** Selected items measuring participant nurses' practice regarding physical restraint use at federally administered hospitals in Addis Ababa, 2019 (*n* = 112) (*n* = 112).

Statements	Always, N (%)	Sometimes, N (%)	Never, N (%)	Mean ± SD
Do you try a few nursing methods before physically restraining the patient?	50 (44.6%)	56 (50%)	6 (5.4%)	1.6 ± 0.59
Before using the PR on the patient, do you find out why you need to do it?	59 (52.7%)	39 (34.8%)	14 (12.5%)	1.6 ± 0.7
Do you respond to the call for help from a restrained patient immediately?	53 (47.3%)	47 (42%)	12 (10.7%)	1.6 ± 0.67
Do you examine restrained patients at least on a two-hour basis?	48 (42.9%)	47 (42%)	17 (15.2%)	1.72 ± 0.7
When giving personal care to the restrained patients, do you examine their skin to find parts which are red or bruised?	64 (57.1%)	36 (32.1%)	12 (10.7%)	1.54 ± 0.68
Did you tell the patients why they are restrained?	45 (40.2%)	56 (50%)	11 (9.8%)	1.7 ± 0.64
Do you tell the family members/visitors why the patient is restrained?	50 (44.6%)	52 (46.4%)	10 (8.9%)	1.64 ± 0.64
Do you restrain patients when you faced a staff shortage?	38 (33.9%)	47 (42%)	27 (24.1%)	1.9 ± 0.75
Do you record the type of restraint, reason, and the time on the card?	34 (30.4%)	41 (36.6%)	37 (33%)	2 ± 0.79
Do you assess the restrained patient frequently to check if the restraint should be removed?	45 (40.2%)	47 (42%)	20 (17.9%)	1.78 ± 0.73
Do you evaluate and record the effect of physical restraint when applied to a patient?	53 (47.3%)	32 (28.6%)	27 (24.1%)	1.77 ± 0.8

**Table 5:** Association of demographic characteristics with participant nurses' knowledge, attitude, and practice in Pearson's correlation coefficient analysis (*n* = 112).

Sociodemographic characteristics		No.	Knowledge score mean ± SD	Attitude score mean ± SD	Practice score
Age in years	20–30	97	5.96 ± 2.67	14.1 ± 3.1	13.7 ± 3.7
	31–40	14	6.78 ± 2	14.3 ± 2.94	15.2 ± 4.2
	>50	1	9	16	17
Pearson' correlation coefficient (*r*)			0.23	0.115	0.216
*P* value			0.015	0.229	0.022
Gender	Male	57	5.8 ± 2.83	14.1 ± 3.6	14 ± 3.8
	Female	55	6.4 ± 2.35	14.2 ± 2.4	13.7 ± 3.8
Pearson' correlation coefficient (*r*)			0.114	0.059	−0.038
*P* value			0.23	0.53	0.6
Marital status	Married	38	6.36 ± 2.31	13.8 ± 3.1	14 ± 3.76
	Unmarried	72	5.94 ± 2.76	14.3 ± 3	13.7 ± 3.8
	Divorced	2	6.5 ± 3.53	15 ± 4.2	17
Pearson' correlation coefficient (*r*)			−0.064	0.031	0.007
*P* value			0.49	0.75	0.9
Work experience	6 months–1 year	20	5.55 ± 2.66	14.5 ± 3.6	13.5 ± 3.9
	2–5 years	70	6.2 ± 2.7	13.9 ± 2.8	14 ± 3.4
	6–10 years	19	6.15 ± 2.24	14.5 ± 3.6	13.5 ± 4.9
	>10 years	3	7 ± 2.64	14 ± 2.6	14 ± 5.2
Pearson' correlation coefficient (*r*)			0.09	0.026	0.033
*P* value			0.34	0.78	0.73
Level of education	Diploma	12	6.16 ± 1.94	15 ± 3.1	14.5 ± 3.2
	Bachelor's degree	94	6 ± 2.73	14.1 ± 3	13.7 ± 3.89
	Master's degree	6	6.8 ± 1.7	13.3 ± 2.4	14.5 ± 3.9
Pearson' correlation coefficient (*r*)			0.031	0.06	−0.021
*P* value			0.74	0.5	0.8
Presence of guideline	Yes		7.3 ± 2.7	14.1 ± 3.4	14.1 ± 3.4
	No		5.5 ± 2.4	13.9 ± 2.8	13.7 ± 3.9
Pearson' correlation coefficient (*r*)			−0.34	−0.052	−0.12
*P* value			0.001	0.5	0.2
Taking training	Yes		7.6 ± 2.4	14.7 ± 3.4	14 ± 3.3
	No		5.4 ± 2.4	13.9 ± 2.9	13.8 ± 4
Pearson' correlation coefficient (*r*)			−0.34	−0.88	−0.03
*P* value			0.001	0.3	0.7

**Table 6:** Factors associated with nurses' practice regarding physical restraint use in bivariate and multivariate analysis (*n* = 112).

Variables		Unsatisfactory practice	Good practice	*P* value	UOR	95% CI		*P* value	AOR (95% CI)
						Lower	Upper		
Age in years	21–30	76	21	0.001	0.3			0.001	0.3
	31–40	7	7	0.029	3.6	1.14	11.5	0.029	3.62 (1.14, 11.5)
	>or = 41	0	1	1					

**Table 7:** Factors associated with nurses' knowledge regarding physical restraint use in bivariate and multivariate analysis (*n* = 112).

Variables		Poor knowledge	Fair knowledge	*P* value	UOR (95% CI)	*P* value	AOR (95% CI)
Training on PR	Trained	11	22	0.061	2	0.6	2.8
	Nontrained	52	21	0.001	0.2 (0.083, 0.49)	0.001	0.2 (0.1, 0.5)
	Do not remember	3	3	0.4	0.5 (0.09, 2.9)	0.44	0.5 (0.09, 2.9)
Written policy and guideline	Have	13	21	0.17	1.62	0.2	
	Do not have	34	17	0.011	0.31 (0.125, 0.76)	0.1	2.5 (0.8, 8)
	They do not know	19	8	0.014	0.26 (0.09, 0.8)	0.6	1.3 (0.4, 3.5)

**Table 8:** Association between practice and knowledge regarding physical restraint use in bivariate analysis (*n* = 112).

Variables		Unsatisfactory practice	Good practice	*P* value	UOR	95% CI		*P* value	AOR (95% CI)
						Lower	Upper		
Knowledge category	Good	5	4	0.064	4	0.9	17.3	0.064	4 (0.9, 17.3
	Fair	23	14	0.019	3.04	1.2	7.7	0.019	3 (1.2, 7.7)
	Poor	55	11	0.001	0.2			0.001	0.2

**Table 9:** Association between attitude and practice regarding physical restraint use in bivariate analysis (*n* = 112).

Variables		Improper attitude	Proper attitude	*P* value	UOR (crude ratio)	95% CI		*P* value	AOR (95% CI)
						Lower	Upper		
Practice category	Unsatisfactory	14	8	0.2	0.57			0.2	
	Satisfactory	21	40	0.02	3.3	1.2	9.2	0.02	3.3 (1.2, 9.2)
	Good	9	20	0.023	3.88	1.2	12.5	0.023	3.9 (1.2, 12.5)

## Data Availability

The data used in this study are available upon reasonable request.

## Abbreviations

AaBET: Addis Ababa Burn, Emergency, and Trauma unit
ALERT: All Africa Leprosy Rehabilitation and Training center
AOR: Adjusted odds ratio
CSA: Central Statistical Agency
ED: Emergency department
HCFA: Health Care Financing Administration
ICU: Intensive-care unit
JCAHO: Joint Commission on Accreditation of Health-Care Organization
MOH: Ministry of Health
NGO: Nongovernmental organization
PDCN: Professional Development Committee of Nursing Council
PR: Physical restraint
SPSS: Statistical package for social sciences
UOR: Unadjusted odds ratio
US: United States
WHO: World Health Organization.
